# Supplementary material for: Displacement Reaction-Assisted Synthesis of Sub-Nanometer Pt/Bi Boost Methanol-Tolerant Fuel Cells
Source: Nanomaterials (Basel). 2022 Apr 11;12(8):1301. doi: 10.3390/nano12081301 (PMC9025715; doi:10.3390/nano12081301)
Supplement: Supplementary file 1 [file nanomaterials-12-01301-s001.zip › nanomaterials-1657039-supplementary.pdf]

Supporting Information

Displacement reaction-assisted synthesis of sub-nanometer Pt/Bi boost methanol-tolerant fuel cells

Xianling Wu[1], Dumei Wang[1], Xueming Kang[1], Dongtang Zhang[1,*], Yong Yan[1], Guangsheng Guo[1,2], Zaicheng Sun[1,*], Xiayan Wang[1]

1 Center of Excellence for Environmental Safety and Biological Effects, Beijing Key Laboratory for Green Catalysis and Separation, Department of Chemistry and Biology, Beijing University of Technology, Beijing 100124, PR China; wuxl@emails.bjut.edu.cn (X.W.); wangdm@emails.bjut.edu.cn (D.W.); kxm@emails.bjut.edu.cn (X.K.); yong.yan@bjut.edu.cn (Y.Y.); gsguo@bjut.edu.cn (G.G.); xiayanwang@bjut.edu.cn (X.W.)

2 Minzu University of China, Beijing 100081, PR China

* Correspondence: zhangdongtang@bjut.edu.cn (D.Z.); sunzc@bjut.edu.cn (Z.S.)

Experimental Section

Reagents and materials: Chloroplatinic acid hexahydrate ($H_2PtCl_6·6H_2O$, 99.9%), bismuth nitrate ($Bi(NO_3)_3·5H_2O$, 99.0%), ethylene glycol (EG， 99%), polyvinylpyrrolidone (PVP, K30, 99.9%) were purchased from Sinopharm Chemical Reagent Beijing Co., Ltd. The borane–ammonia complex ($NH_3·BH_3$, 97%), commercial Pt/C (Pt/C, 20 wt %), and Nafion 117 solution (5%) were purchased from Sigma-Aldrich. Reduced graphene oxide (rGO) was purchased from the graphene supermarket. The ultrapure water was used in the experiment (18.2 MΩ cm).

Instrumentation: The X-ray diffraction (XRD) patterns for the samples were obtained using a Bruker D8 Advance diffractometer with Cu-Kα (λ=1.5405 Å) radiation source (40 kV, 40 mA). Transmission electron microscopy (TEM) was carried out with a JEOL JEM-2100 microscope operating at 200 kV with a nominal resolution. The composition of the prepared catalysts was measured using an inductively coupled plasma-atomic emission spectrometer (ICP-AES) on an IRIS Intrepid spectrometer after the dissolution of the samples in aqua regia.

Preparation of ultra-small PtBi/rGO catalyst. First, 0.1 mmol of $Bi(NO_3)_3·5H_2O$ and 167 mg of PVP were dissolved in 50 mL of EG confined in a beaker under constant magnetic stirring. Then, 10 mg of rGO powder was added and well-distributed using an ultrasonic cleaning bath. Next, 6 mg $NH_3·BH_3$ was added to that solution and 0.05 mmol H_2PtCl_6 was added after 30 min. Subsequently, the solution and the mixture were stirred for 12 h. The final products were collected by a vacuum filtration process and then washed successively with ethanol. As identified below, the produced catalyst sizes

at the atom level were obtained. Because of the addition of graphene to form the supported catalyst, the catalyst should be expressed as PtBi/graphene.

Electrochemical measurement

The electrochemical measurements were performed on a CHI 1030C at room temperature. The glassy carbon rotating disk electrode (GC-RDE, 4 mm) was used as the working electrode. A GC film electrode was used as the counter electrode and the reference electrode. All the potentials in this study are presented with reference to Ag/AgCl. Before using the GC electrode as a substrate for the catalysts, it was polished with 0.05 mm alumina to yield a mirror finish. The catalysts were re-dispersed in ethanol and 50 μL Nafion (0.5 wt%) (the concentration of Pt is 1.0 mg mL^{-1}, dilute the solution according to the ICP quantitative results to obtain the final solution) under ultrasonication for 30 min. The working electrode was prepared by transferring 10 μL of the catalyst ink on the surface of GC-RDE and drying it in the air. Cyclic voltammetry (CV) measurements were carried out in N_2-saturated 0.5 mol/L H_2SO_4 solutions at 50 mV/s. The ORR measurements were performed in O_2-saturated 0.5 mol/L H_2SO_4 solutions using GC-RDE at a sweep rate of 10 mV/s. In the ORR polarization curve, the current densities were normalized with reference to the Pt mass loading on the GC-RDE.

Before measurement, the working electrode loaded with the as-prepared catalysts was electrochemically cleaned by continuous potential cycling between 0.0 and 1.2 V (vs. RHE) at 200 mV/s in 0.10 mol/L $HClO_4$ solution until stable cyclic voltammetry (CV) curve was obtained. The ORR polarization measurements were performed in O_2-

saturated 0.1 mol/L $HClO_4$ solutions using GC-RDE at a sweep rate of 10 mV/s under different rotation rates. The durability test was performed at room temperature in O_2-saturated 0.1 mol/L $HClO_4$ by the chronoamperometry (*I–t*) experiments at 1600 rpm. The potential vs. Ag/AgCl reported in the manuscript were converted to the RHE according to the following equation:

Potential (V vs. RHE) = Applied potential (V vs. Ag/AgCl/sat. KCl) + 0.199 V + 0.0592 × pH.

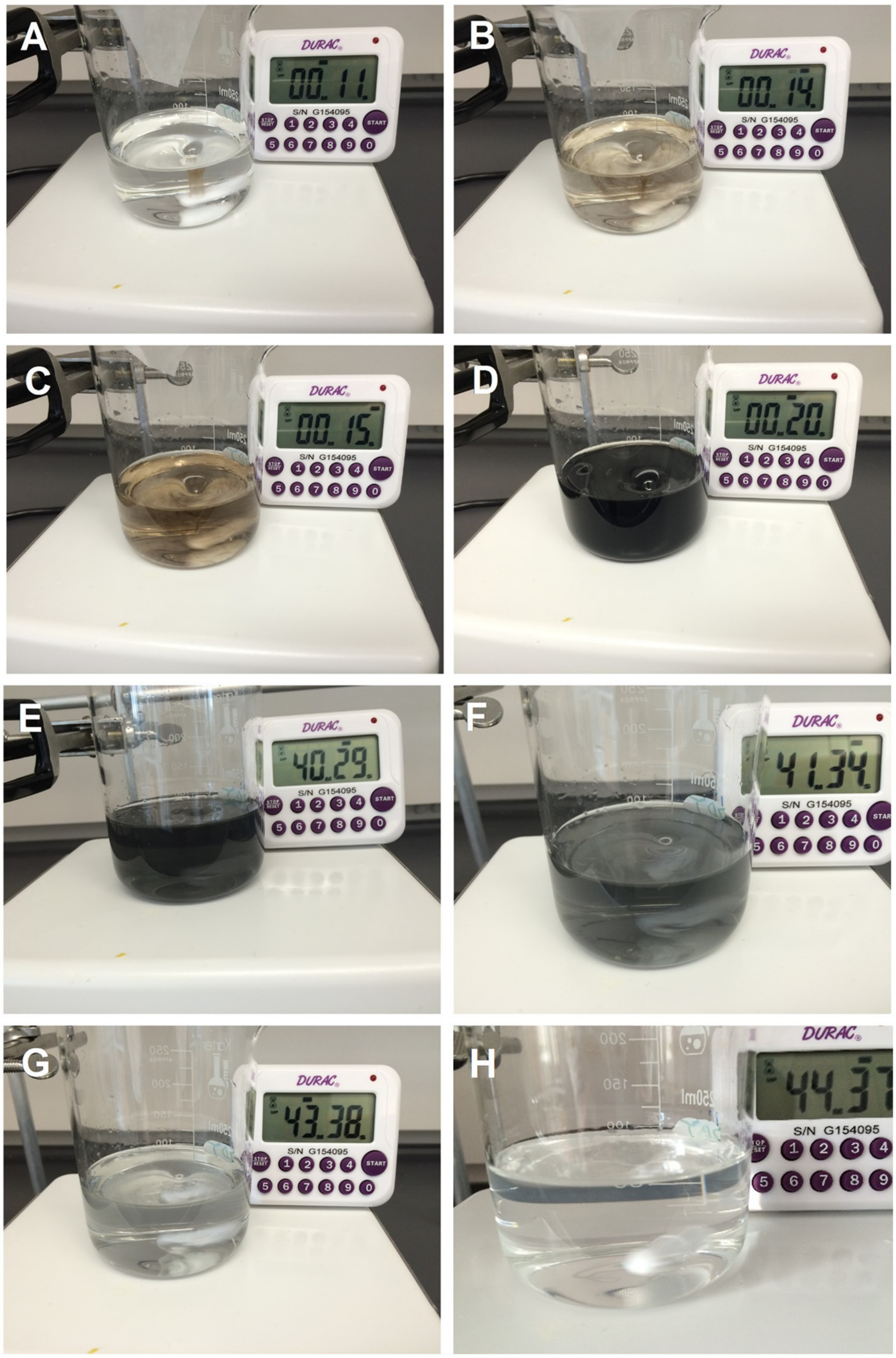

Figure S1. Photographs of the experimental process of $Bi(NO_3)_3$ reduction with NH_3BH_3 without carbon support.

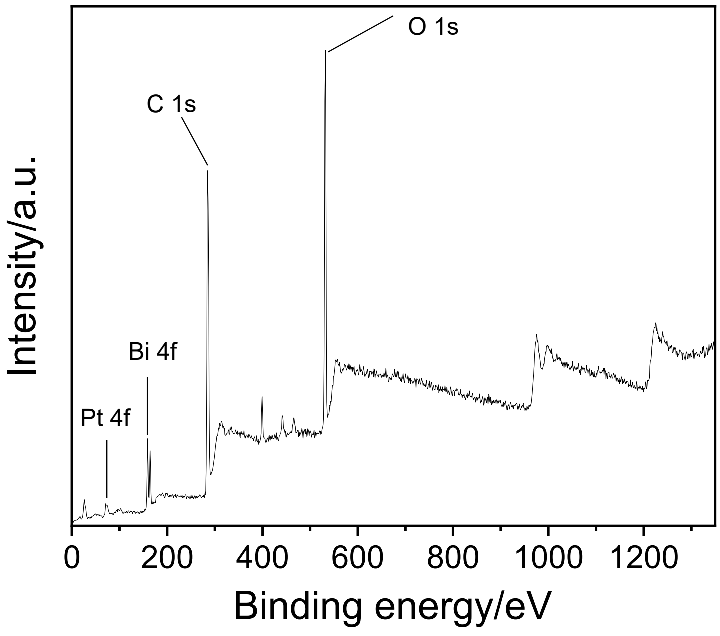

Figure S2. The full XPS spectra of Pt/Bi/rGO.

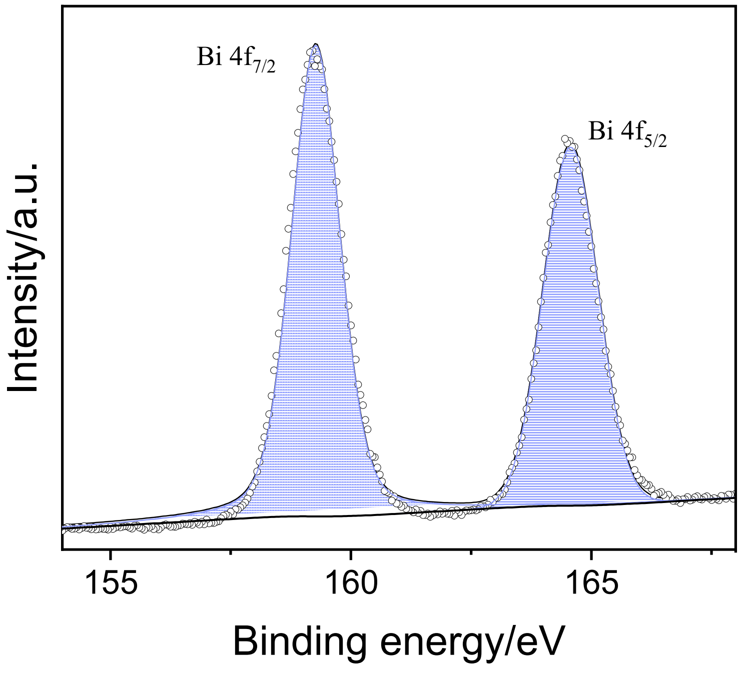

Figure S3. High-resolution XPS spectra of Bi in Pt/Bi/rGO.

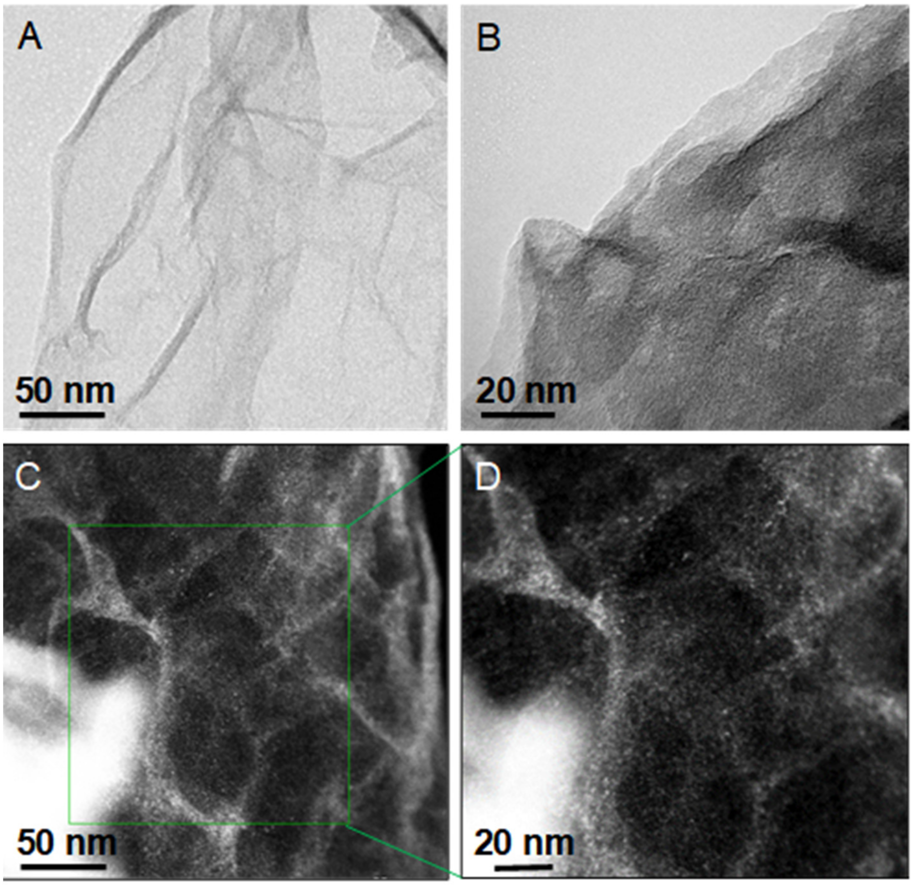

Figure S4. TEM images (A, B) and HAADF images (C, D) of ultra-small PtBi/C at different magnifications

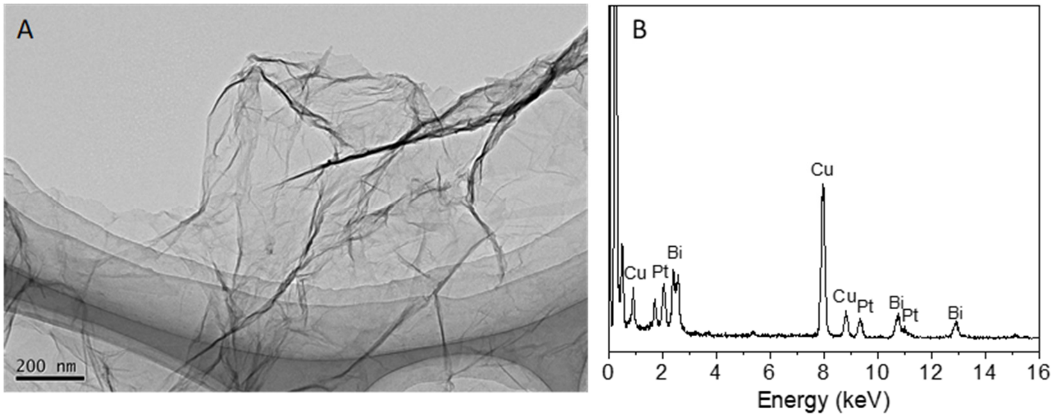

Figure S5. TEM image (A) and EDS spectrum analysis (B) of ultra-small PtBi-rGO.

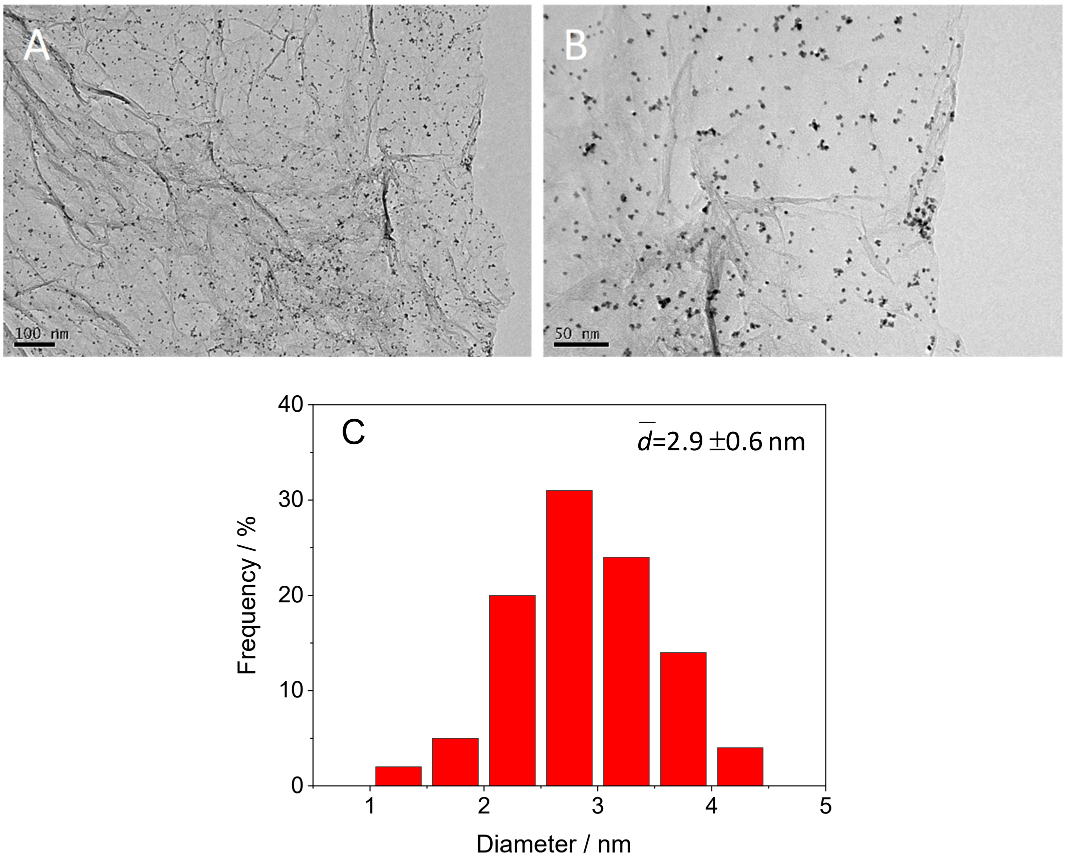

Figure S6. TEM images of Pt/rGO obtained without $Bi(NO_3)_3$ during synthesis (A, B) and size histogram of Pt. The average particle diameter of 2.9±0.6 nm and a size distribution range of 1.34–4.32 nm (C).

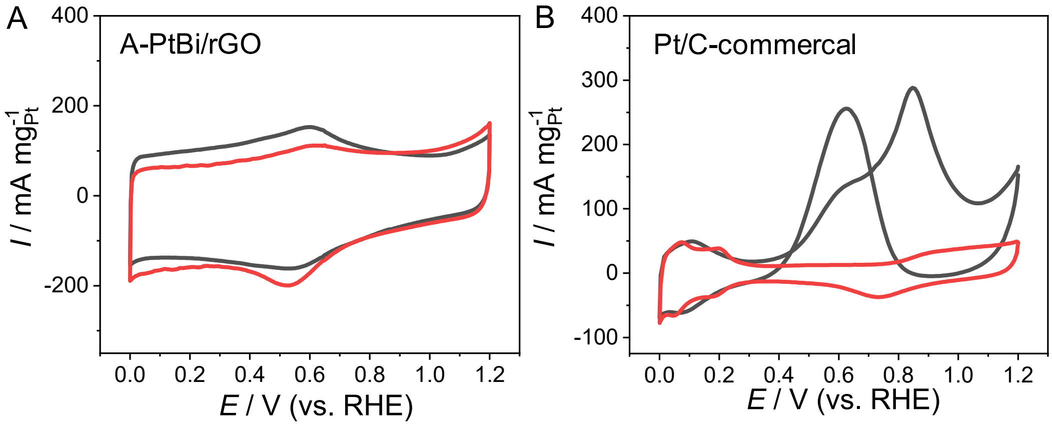

Figure S7. CV curves measured in 0.5 mol/L H_2SO_4 and 0.5 mol/L methanol aqueous solution for the ultra-small PtBi/graphene catalysts (A) and commercial Pt/C (B), respectively.

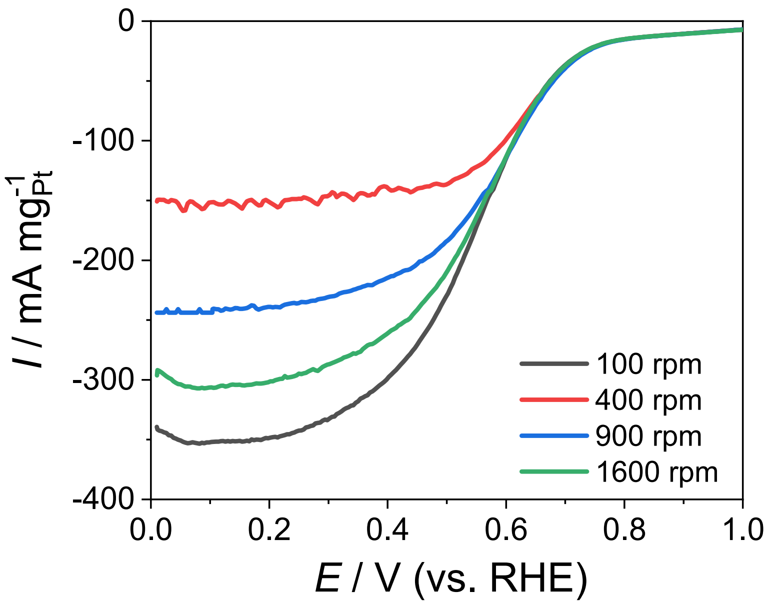

Figure S8. Polarization curves for the ORR with ultra-small PtBi/graphene catalysts at 10 mV/s in oxygen-saturated 0.5 mol/L H_2SO_4.

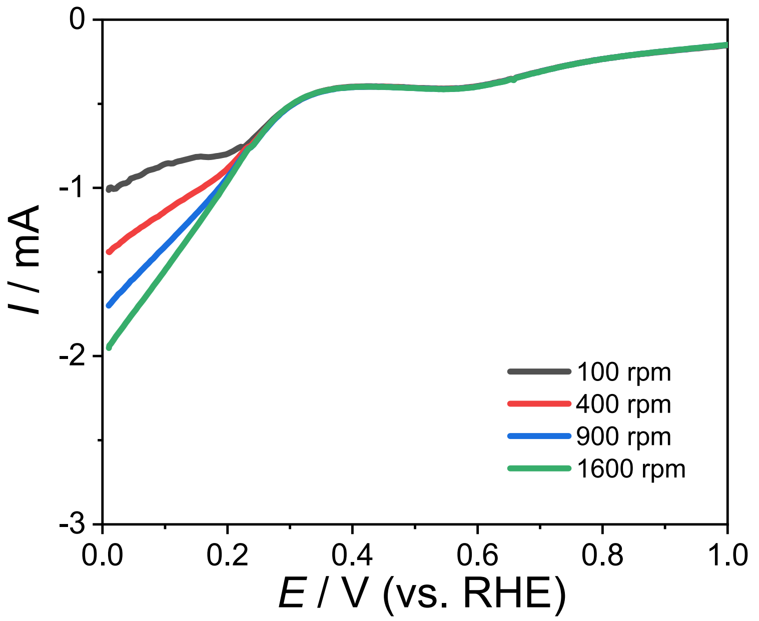

Figure S9. Polarization curves for the ORR on rGO at 10 mV/s in oxygen-saturated 0.5 mol/L H_2SO_4.

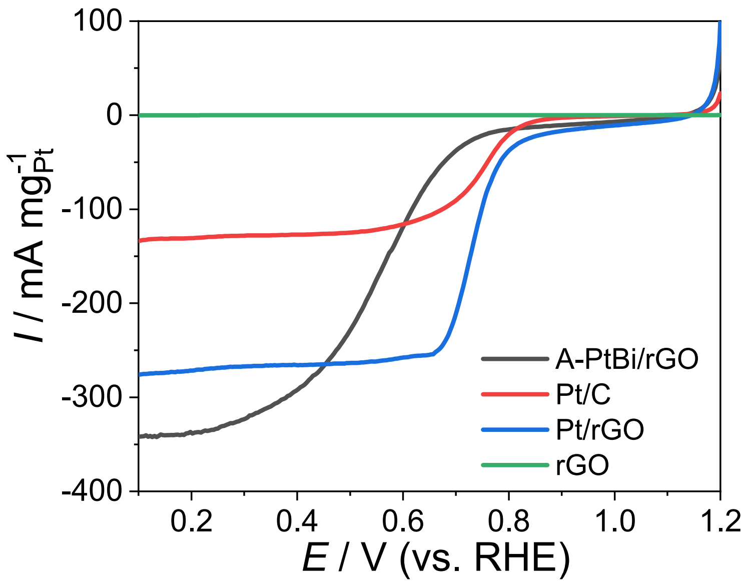

Figure S10. Polarization curves for the ORR on ultra-small PtBi/graphene, Pt/C, and Pt/graphene catalysts at 10 mV/s in oxygen-saturated 0.5 mol/L H_2SO_4.
